# Subcutaneous injection of IFN alpha-2b for COVID-19: an observational study

**DOI:** 10.1186/s12879-020-05425-5

**Published:** 2020-10-02

**Authors:** Bo Wang, Diandian Li, Tao Liu, Haohua Wang, Fengming Luo, Yanbin Liu

**Affiliations:** 1 Department of Respiratory and Critical Care Medicine, West China Hospital, Sichuan University, Chengdu, 610041 Sichuan China; 2 The Red Cross Hospital of Wuhan (The Eleventh Hospital of Wuhan City), Wuhan, 430015 Hubei China; 3 West China School of Medicine, Sichuan University, Chengdu, 610041 China; 4 Center of Infectious Diseases, West China Hospital, Sichuan University, Chengdu, 610041 Sichuan China

**Keywords:** Interferon alpha-2b, Subcutaneous injection, Viral clearance, COVID-19

## Abstract

**Background:**

The global pandemic of coronavirus disease 2019 (COVID-19) infection is ongoing and associated with high mortality. The aim of this study was to investigate the efficacy and safety of subcutaneous injection of interferon alpha-2b (IFN alpha-2b) combined with lopinavir/ritonavir (LPV/r) in the treatment of COVID-19 infection, compared with that of using LPV/r alone.

**Methods:**

Patients diagnosed with laboratory-confirmed COVID-19 infection in Wuhan Red Cross hospital during the period from January 23, 2020 to March 19, 2020 were included. The length of stay, the time to viral clearance and adverse reactions during hospitalization were compared between patients using oral LPV/r and combined therapy of LPV/r and subcutaneous injection of IFN alpha-2b.

**Results:**

A total of 22 patients were treated with LPV/r alone and 19 with combined therapy with subcutaneous injection of IFN alpha-2b. The average length of hospitalization in the combination group was shorter than that of LPV/r group (16 ± 9.7 vs 23 ± 10.5 days; *P* = 0.028). Moreover, the days of hospitalization in early intervention group decreased from 25 ± 8.5 days to 10 ± 2.9 days compared with delayed intervention group (*P* = 0.001). Combined therapy with IFN alpha-2b also significantly reduced the duration of detectable virus in the upper respiratory tract. No patient in each group was transferred to intensive care unit (ICU) or died during the treatment. There was no significant difference in the adverse effect composition between two groups.

**Conclusions:**

Subcutaneous injection of IFN alpha-2b combined with LPV/r shortened the length of hospitalization and accelerated viral clearance in COVID-19 patients, which deserves further investigation in clinical practice.

## Background

Since December 2019, large numbers of patients were diagnosed with severe acute respiratory syndrome coronavirus 2 (SARS-CoV-2) infections worldwide, also known as coronavirus disease 2019 (COVID-19), with a rapid growth rate exceeding that of “severe acute respiratory syndrome” in 2003. As of June 10, 2020, over 7 million COVID-19 cases and 406,668 deaths have been confirmed globally. SARS-CoV-2 infection can lead to intensive care unit (ICU) admission and high mortality [1]. It is still a challenge to effectively shorten the duration of viral shedding and the length of hospitalization, so as to avoid a run on local medical resources, reduce the occurrence of severe complications and improve the recovery rate. In the absence of a SARS-CoV-2 specific vaccine, finding an effective antiviral therapy becomes crucial.

Type I interferons have broad spectrum antiviral activities against RNA viruses. They are approved to treat hepatitis B and C but it is also reported to inhibit SARS-CoV reproduction in vitro [2]. According to the seventh edition of the Chinese Guidelines, in addition to oral medication such as lopinavir/ritonavir (LPV/r) and ribavirin, vapor inhalation of IFN alpha may be considered. Clinical trials have been registered to test whether therapeutic regimens of IFN alpha-2b combined with LPV/r can be beneficial for the treatment of COVID-19. The method for administration of IFN alpha is vapor inhalation at a dose of 5 million U (and 2 mL of sterile water for injection) for adults, 2 times/day [3, 4]. The aerosols produced during treatment with atomized IFN alpha are also exhaled, influencing its efficacy. Moreover, whether vapor inhalation of the medication has a systemic therapeutic effect for treating COVID-19 remains unclear.

Herein, we performed a retrospective cohort study in patients with confirmed COVID-19 on the efficacy and safety of subcutaneous injection of IFN alpha-2b combined with LPV/r, compared with that of using LPV/r alone.

## Methods

### Subjects and study design

The research protocol of this single-center, retrospective cohort study conformed to the principles of the Declaration of Helsinki and was approved by the institutional ethics board of West China Hospital of Sichuan University (No.2020–126). Written informed consent was collected from all patients. Patients diagnosed with laboratory-confirmed COVID-19 infection in Wuhan Red Cross hospital during the period from January 23, 2020 to March 19, 2020 were included. Nasopharyngeal swabs of upper respiratory tract were collected from all patients by two investigators (Tao Liu and Yanbin Liu), and patients enrolled in this study were diagnosed according to the following criteria based on WHO recommendation: isolation of COVID-19 or at least two positive results by reverse-transcription-polymerase-chain-reaction (RT-PCR) assay for COVID-19 or a genetic sequence that matched COVID-19 [5]. Eligible patients were those aged 18 years or older. Patients who were intubated, who died, or who were transferred to another hospital within 24 h after admission were excluded. Patients receiving oral LPV/r were classified as LPV/r group, while those given LPV/r and subcutaneous injection of IFN alpha-2b were assigned into combination group.

### Procedures

Treatments during the epidemic were empirical. All eligible patients rested in bed in the isolation wards and daily sufficient caloric intake, balance of water-electrolyte and stability of internal environment were ensured. Oxygen therapy was given according to each patient’s oxygen saturation. Antibiotics were only administered in patients with combined bacterial infection. For anti-viral treatment, LPV/r group were given oral lopinavir/ritonavir tablets (Abb-Vie Ltd., North Chicago, IL, USA; 200 mg/50 mg/ pill), 400 mg/ time, twice a day, and combination group were supplemented with subcutaneous injection of IFN alpha-2b (3SBIO Inc., Shenyang, China; 3 million IU/ dose), 3 million IU/time, qod, in addition to LPV/r treatment. The course of LPV/r treatment was 10 days, while IFN was used until the virus was detected negative by RT-PCR in two consecutive respiratory specimens (≥1 day apart). In addition to regular clinical monitoring (body temperature, respiratory rate, blood pressure, pulse, symptoms and signs), blood count, liver enzymes and renal function were assessed at baseline and throughout the treatment course. Two investigators (Bo Wang and Yanbin Liu) obtained all clinical information, including demographic data, medical history, co-morbidities symptoms, signs, laboratory findings and management from electronic medical records of Wuhan Red-cross Hospital.

### Outcome measurements

The primary outcomes were the length of hospitalization and the time to viral clearance of SARS-CoV-2 in the upper respiratory tract from hospital admission. The secondary outcomes included adverse effects, ICU admission rate, and hospital mortality during the treatment.

### Statistical analysis

Continuous variables were presented as mean ± standard deviation (SD), while categorical variables were presented as counts and percentages. Means for continuous variables were compared using independent *t* test when the data were normally distributed; otherwise, the Mann-Whitney test was used. Proportions for categorical variables were compared using the χ2 test or Fisher-Exact test when appropriate. Time to viral clearance, defined as the days from hospital admission to the first negative PCR of two negative consecutive PCR tests, was portrayed by Kaplan-Meier plot and compared with a log-rank test. *P* < 0.05 was considered statistically significant. All statistical analyses were performed by SPSS 25.0 for Windows (IBM, Chicago, IL, USA).

## Results

### Patient characteristics

A total of 41 patients older than 18 years fulfilling the WHO criteria of SARS-CoV-2 infection were enrolled in this study. The average age was 60.6 years and 18 (43.9%) were male. Twenty-four patients had one or more coexisting medical conditions, including hypertension, diabetes, hepatitis and malignancy. twenty-two patients received oral LPV/r only and 19 were given combined therapy of oral LPV/r and subcutaneous injection of IFN alpha-2b. Baseline characteristics including age, gender, comorbidities, and time from onset to admission were generally similar between the two groups (*P* > 0.05; Table 1).

**Table 1:** Baseline characteristics of patients with COVID-19

	LPV/r group (*n* = 22)	Combination group (*n* = 19)	*P*-value
Age, year (mean ± SD)	62.0 ± 9.4	56.2 ± 9.7	0.058
Male, n (%)	8 (36)	10 (53)	0.295
Days from symptom onset to hospital admission (mean ± SD)	11 ± 6.8	12 ± 7.3	0.214
Coexisting medical conditions, n (%)			
Hypertension	5 (22.7)	4 (21.1)	1.000
Diabetes	5 (22.7)	6 (31.6)	0.524
Hepatitis	1 (4.5)	0 (0)	1.000
Malignancy	1 (4.5)	2 (10.5)	0.895
Symptoms, n (%)			
Cough	13 (59)	10 (52.6)	0.678
Shortness of breath	6 (27.3)	4 (21.1)	0.922
Pharyngalgia	2 (9)	4 (21.1)	0.524
Arthralgia/myalgia	7 (31.8)	4 (21.1)	0.438
Chest pain	4 (18.2)	2 (10.5)	0.804
Fatigue	7 (31.8)	9 (47.4)	0.309

### Primary outcomes

**Fig. 1:**
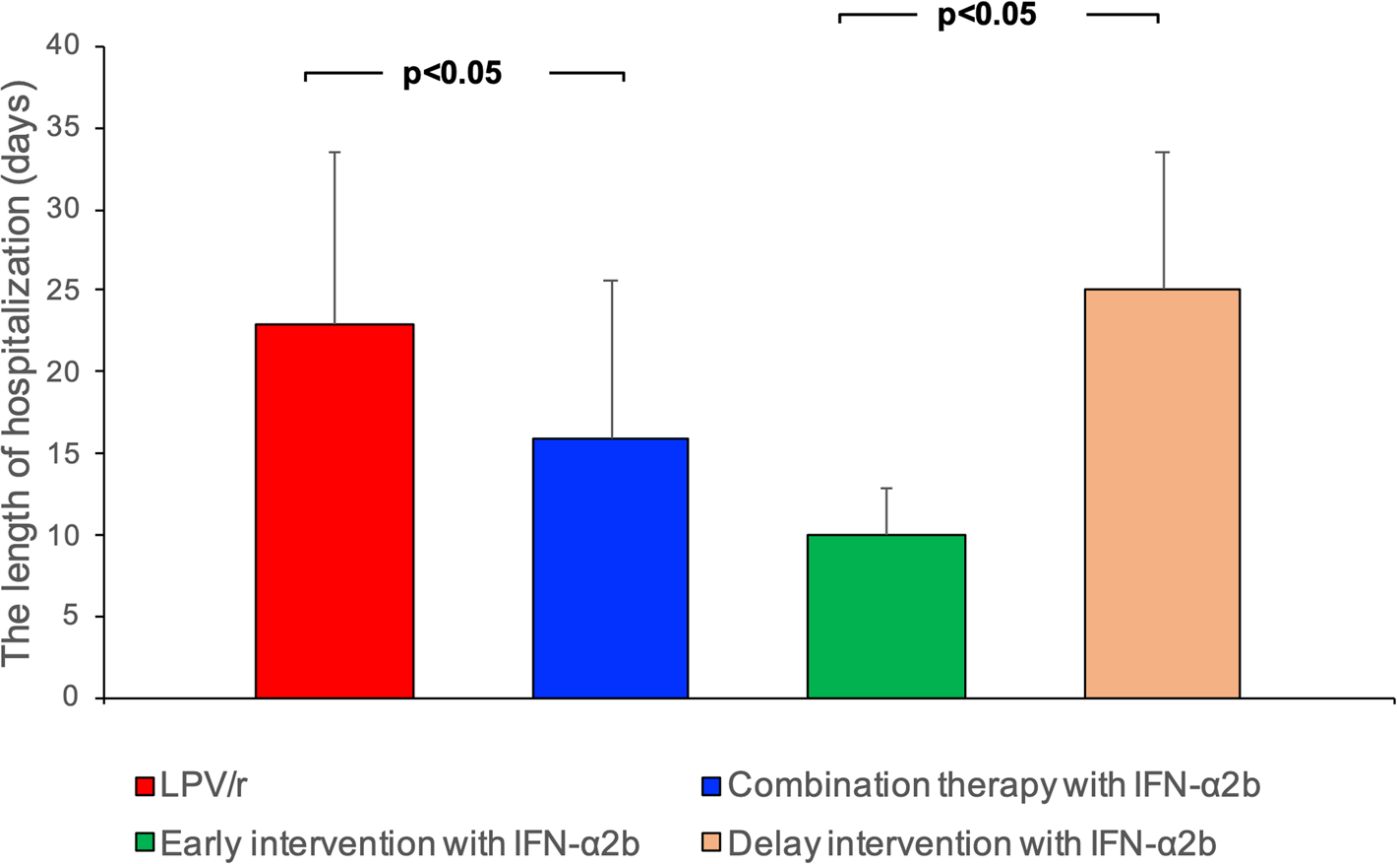
Comparison of length of hospitalization among patients with COVID-19 by patient group. Confirmed COVID-19 patients were treated with oral LPV/r alone or combined therapy of subcutaneous IFN alpha-2b injection and oral LPV/r. For combination group, patients who received IFN alpha-2b within 72 h of admission were defined as the early intervention group, while those who received IFN alpha-2b after 72 h of admission were defined as the delayed intervention group. Data are expressed as mean ± SD

**Fig. 2:**
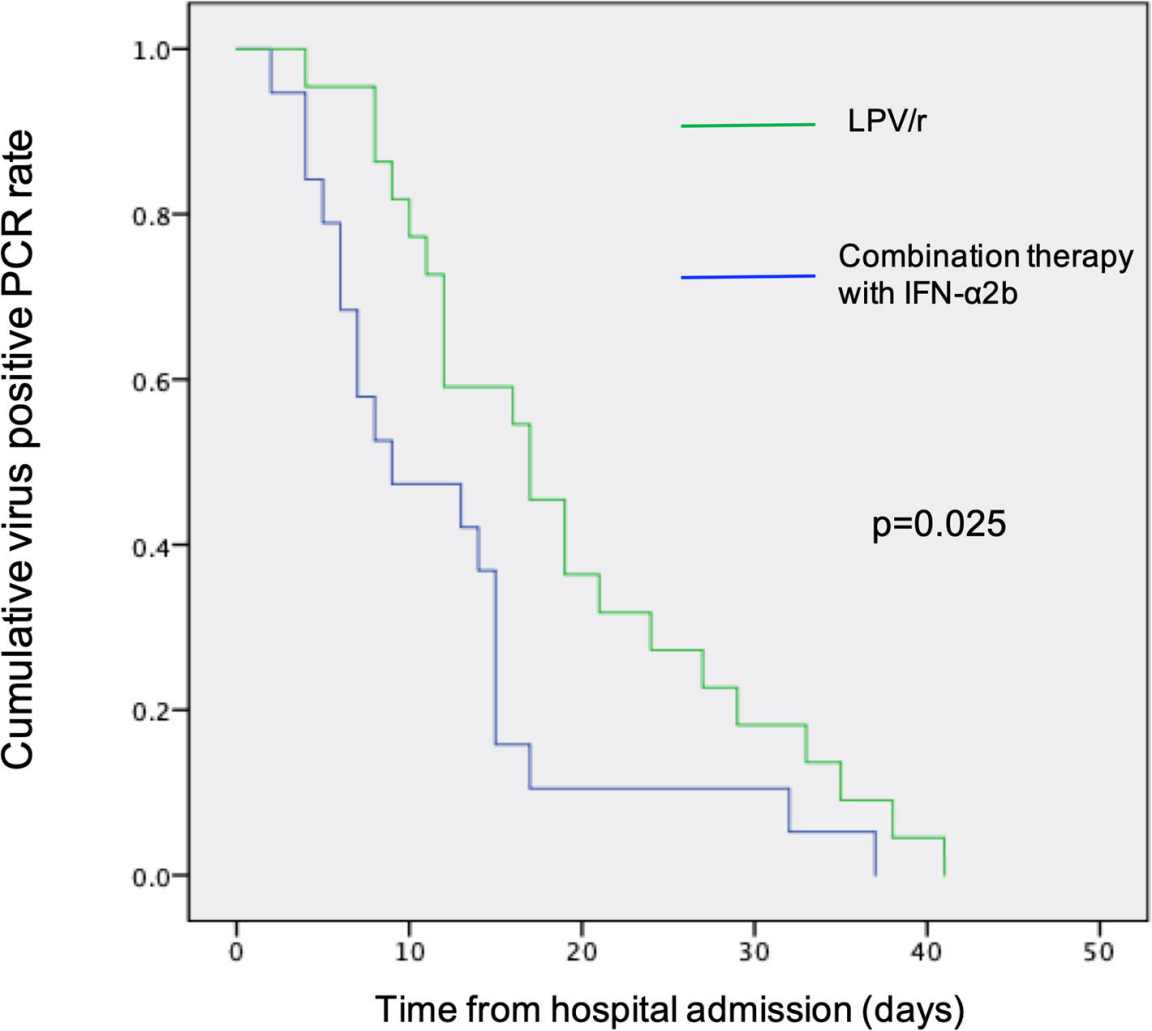
Subcutaneous injection of IFN alpha-2b accelerated viral clearance. Time to viral clearance of SARS-CoV-2 by RT-PCR among patients with COVID-19 by patient group

The average length of hospitalization was 20 days (range 5–45). As shown in Fig. 1, the average length of hospitalization in combination group was significantly shorter than that of LPV/r group (16 ± 9.7 vs 23 ± 10.5 days; *P* = 0.028). Further comparison was conducted between early intervention (11 patients received IFN alpha-2b within 72 h of admission) and delayed intervention (8 patients received IFN alpha-2b after 72 h of admission) with IFN alpha-2b, which indicated that the days of hospitalization in early intervention group decreased from 25 ± 8.5 days to 10 ± 2.9 days compared with delayed intervention group (*P* = 0.001). As shown in Fig. 2, assessing the time to viral clearance, the data revealed a significantly accelerated viral clearance in patients receiving combined therapy of oral LPV/r and subcutaneous injection of IFN alpha-2b (*P* < 0.05).

### Secondary outcomes

Transient fever (maximum at 38.0 °C) and digestive upsets were present in 4 and 11 patients, respectively. Six patients showed decreased white blood cell count, while 11 patients had elevated transaminase. No patient in each group was transferred to ICU or died during the treatment. There was no significant difference in the adverse effect composition between two groups (*P* > 0.05; Table 2). All adverse effects were alleviated after symptomatic treatment, and no anti-viral treatment was discontinued.

**Table 2:** Adverse effects in patients with COVID-19 by patient group

	LPV/r group (*n* = 22)	Combination group (*n* = 19)	*P*-value
Decreased white blood cell count, n (%)	3 (13.6)	3 (15.8)	1.000
Elevated transaminase, n (%)	6 (27.3)	5 (26.3)	0.945
Mild anemia, n (%)	2 (9)	0 (0)	0.490
Transient fever, n (%)	1 (4.5)	3 (15.8)	0.495
Digestive upsets, n (%)	6 (27.3)	5 (26.3)	0.945

## Discussion

By inducing an antiviral response across a wide range of cell types and mediating adaptive immune response, type I IFNs can interfere with the replication and spread of virus [6]. Clinically, type I IFNs have already been approved for use in the treatment of viral infections (hepatitis B and hepatitis C), autoimmune disorders and certain cancers. Importantly, treatment of type I IFNs has been studied against SARS-CoV and MERS-CoV in numerous in vitro and in vivo experiments, in combination with or not with lopinavir/ritonavir, ribavirin and corticosteroids [7–9]. The knowledge gained from these studies may be valuable in the selection of potential treatments against SARS-CoV-2, since MERS-CoV and SARS-CoV share some similar properties with SARS-CoV-2 [10]. SARS-CoV-2 displays in vitro a substantial sensitivity to IFN alpha pretreatment, implying that IFN alpha might be used as a prophylaxis against SARS-CoV-2 [11].

More recently, analyses have suggested that inhaled IFN alpha-2b accelerated viral clearance from the respiratory tract and hastened resolution of systemic inflammatory processes when compared to arbidol treatment alone [12]. The administration of IFN alpha-2b by vapor inhalation may offer the advantage of targeting specifically the respiratory tract, but the pharmacodynamics and pharmacokinetics of this mode need further assessment. Notably, SARS-CoV-2 has been observed to have an organotropism beyond the respiratory tract, including the kidneys, liver, heart, and brain, which could possibly influence the course of COVID-19 disease and aggravate preexisting conditions [13]. Systemic therapeutic effect of vapor inhalation of IFN alpha-2b remains unclear. Besides, vapor inhalation may also promote the formation of aerosols, increasing the risk of aerosol transmission of virus in a relatively closed environment.

On the contrary, the subcutaneous and intravenous modes of administration have been well-described and already proven safe in several clinical trials. However, the efficacy and safety of subcutaneous injection of IFN alpha have not been evaluated in SARS-CoV-2 infection. Hung et al. reported that triple combination of IFN beta-1b, LPV/r and ribavirin antiviral therapy was safe and superior to LPV/r alone in shortening the duration of viral shedding and hospital stay in patients with COVID-19 [14]. The research also noted that a double antiviral therapy with interferon as a backbone was warranted. In current study, we found that subcutaneous administration of IFN alpha-2b appeared to shorten the length of hospitalization and the duration of viral shedding. Moreover, among 11 patients who received IFN alpha-2b within 72 h of admission, the average length of stay was 10 days, which was far shorter than that of patients given IFN alpha-2b after 72 h of admission. These results were consistent with previous studies on MERS-CoV showing that a delay in starting treatment was associated with worse outcomes [15], suggesting that earlier subcutaneous injection of IFN alpha-2b may be associated with a higher efficacy in the treatment of SARS-CoV-2 infection. In addition, our study found that supplementary of IFN alpha-2b was adequately tolerated and no new safety concerns were identified. The overall proportion of patients with adverse events were similar between two groups.

Our study has several limitations due to the small sample size and its retrospective, nonrandomized nature. Meanwhile, it impossible to show any association between temporal viral load changes and antiviral therapy due to the absence of serial viral load measurement in upper and lower respiratory tract specimens. Besides, although baseline characteristics of the two groups seem to be balanced, the average age of IFN group was almost 6 years younger than that of control group (*P* = 0.058), which might overestimate the efficacy of IFN. Impact of other confounders on the course of COVID-19 disease should be reassessed in larger cohorts, and selection and unmeasured confounding bias cannot be excluded. Therefore, we could not easily draw an accurate conclusion about the role of IFN alpha-2b in patients with COVID-19 by now.

## Conclusions

Taken together, our clinical experience and available descriptive data from the therapeutic process are prone to support subcutaneous injection of IFN alpha-2b for specific subgroup of COVID-19 patients. However, large-scale randomized controlled trials are needed to look at the effectiveness, as well as the appropriate dosages and timing of subcutaneous injection of IFN alpha-2b in COVID-19.

## Data Availability

The data that support the findings of this study are available from the corresponding author upon reasonable request.

## Abbreviations

IFN: Interferon
COVID-19: Coronavirus disease 2019
LPV/r: Lopinavir/ritonavir
ICU: Intensive care unit
SARS-CoV-2: Severe acute respiratory syndrome coronavirus 2
RT-PCR: Reverse-transcription-polymerase-chain-reaction
SD: Standard deviation
